# Author Correction: DNA damage and somatic mutations in mammalian cells after irradiation with a nail polish dryer

**DOI:** 10.1038/s41467-023-37245-x

**Published:** 2023-03-14

**Authors:** Maria Zhivagui, Areebah Hoda, Noelia Valenzuela, Yi-Yu Yeh, Jason Dai, Yudou He, Shuvro P. Nandi, Burcak Otlu, Bennett Van Houten, Ludmil B. Alexandrov

**Affiliations:** 1 Department of Cellular and Molecular Medicine, UC San Diego, La Jolla, CA 92093 USA; 2 Department of Bioengineering, UC San Diego, La Jolla, CA 92093 USA; 3 Moores Cancer Center, UC San Diego, La Jolla, CA 92037 USA; 4 UPMC-Hillman Cancer Center, University of Pittsburgh, Pittsburgh, PA 15213 USA

**Keywords:** Cancer, Cancer genomics

Correction to: *Nature Communications* 10.1038/s41467-023-35876-8, published online 17 January 2023

The original version of this Article contained an error in the Abstract, which incorrectly read ‘However, no experimental evaluation has been conducted to reveal the effect of radiation emitted by UV-nail polish dryers on mammalian cells.’ The correct version replaces this sentence with ‘However, the effect of radiation emitted by UV-nail polish dryers on the physiology and mutagenesis of mammalian cells remains unclear.’. This has been corrected in both the PDF and HTML versions of the Article.

