# Supplementary material for: DNA damage and somatic mutations in mammalian cells after irradiation with a nail polish dryer
Source: Nat Commun. 2023 Jan 17;14:276. doi: 10.1038/s41467-023-35876-8 (PMC9845303; doi:10.1038/s41467-023-35876-8)
Supplement: Supplementary file 1 — Supplementary Information [file 41467_2023_35876_MOESM1_ESM.pdf]

SUPPLEMENTARY INFORMATION

DNA damage and somatic mutations in mammalian cells after irradiation with a nail polish dryer

Maria Zhivagui[1-3], Areebah Hoda[1], Noelia Valenzuela[3], Yi-Yu Yeh[2], Jason Dai[2], Yudou He[1-3], Shuvro P. Nandi[1-3], Burcak Otlu[1-3], Bennett Van Houten[4], and Ludmil B. Alexandrov[1-3*]

Affiliations

[1]Department of Cellular and Molecular Medicine, UC San Diego, La Jolla, CA, 92093, USA

[2]Department of Bioengineering, UC San Diego, La Jolla, CA, 92093, USA

[3]Moores Cancer Center, UC San Diego, La Jolla, CA, 92037, USA

[4]UPMC-Hillman Cancer Center, University of Pittsburgh, PA 15213

[*]Correspondence should be addressed to L2alexandrov@health.ucsd.edu.

Table of Contents

Supplementary Figure 1. Exposure and barrier bypass-clonal expansion assays...................3

Supplementary Figure 2. Evaluation of cyclobutane pyrimidine dimers and 6-4 photoproducts irradiated by a UV-nail dryer...5

Supplementary Figure 3. Cytosolic and extra-cellular measurement of reactive oxygen species in MEFs and HFFs...7

Supplementary Figure 4. Mitochondrial membrane potential after irradiation with a UV-nail dryer. 9

Supplementary Figure 5. Schematic of whole-genome data analysis. 11

Supplementary Figure 6. Analysis of variant allelic frequency analysis...............................12

Supplementary Figure 7. Mutation analysis of UV-treated MEF and HFF clones and PCAWG samples. 13

Supplementary Figure 8. Toxicity and characteristics of the UV-nail polish dryer machine. 14

Supplementary Table 1. Assays and timepoints for assessing DNA damage.........................15

SUPPLEMENTARY FIGURES

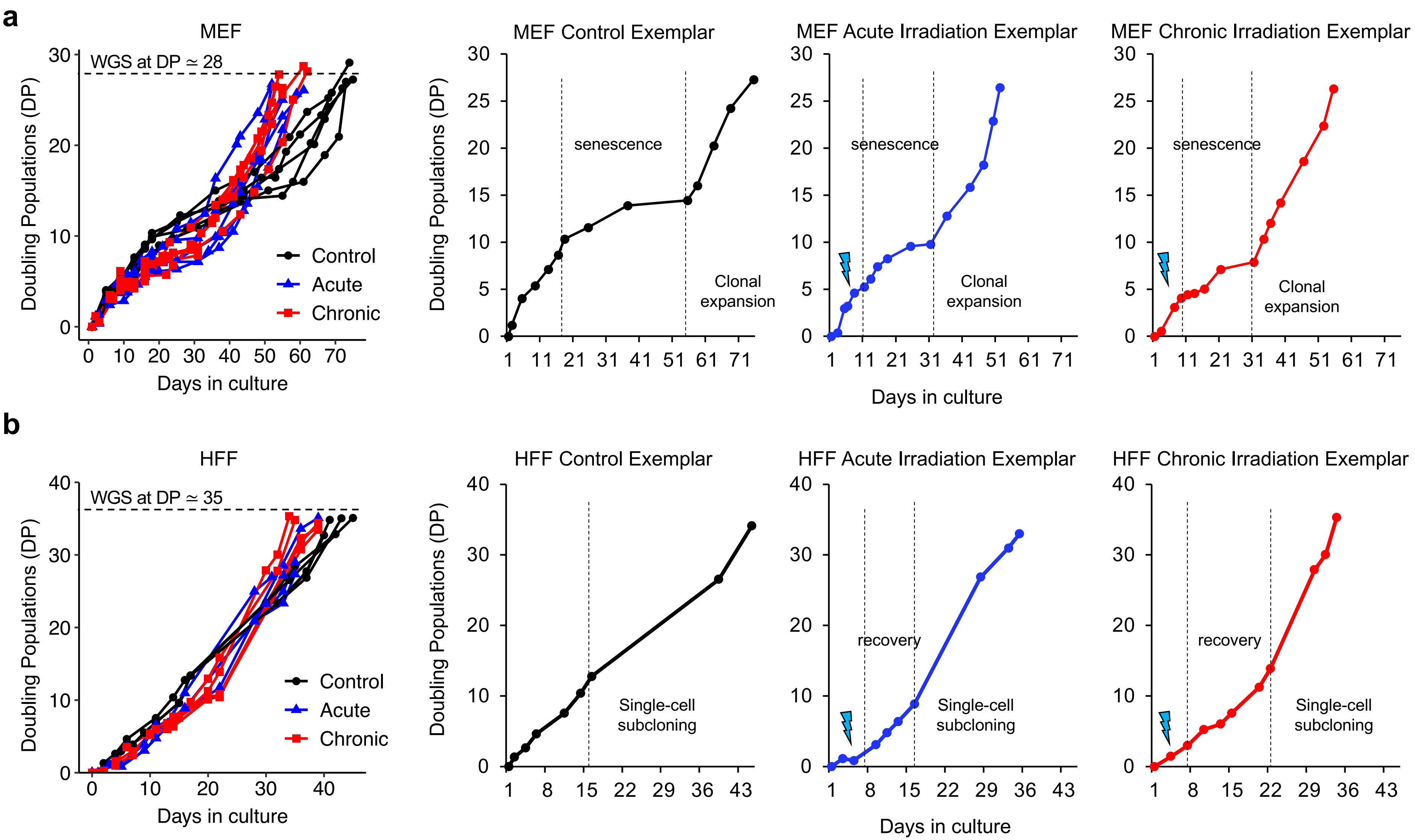

Supplementary Figure 1. Exposure and barrier bypass-clonal expansion assays. ***(a)*** MEFs were treated at early passages and led through senescence manifested by a plateau-like curve where cells lost the ability to efficiently duplicate and grow. Cells went through senescence bypass and

eventually emerge into immortalized clones, able to multiply indefinitely, a process called barrier-bypass clonal expansion, emulating oncogenesis. Irradiated and control MEF cells were grown for ~28 doubling populations prior to bulk whole-genome sequencing (WGS). Exemplars are provided for one control, one acutely irradiated, and one chronically irradiated MEF. Y-axes reflect the number of doubling populations, while X-axes correspond to the number of days in culture. ***(b)*** HFFs were treated at early passages and led through recovery phase before initiating a single cell subcloning assay using a serial dilutions technic. *Bona fide* single-cell clones were collected at the end of experiment. Irradiated and control HEF cells were grown for ~35 doubling populations prior to bulk whole-genome sequencing (WGS). Exemplars are provided for one control, one acutely irradiated, and one chronically irradiated HFF. Y-axes reflect the number of doubling populations, while X-axes correspond to the number of days in culture.

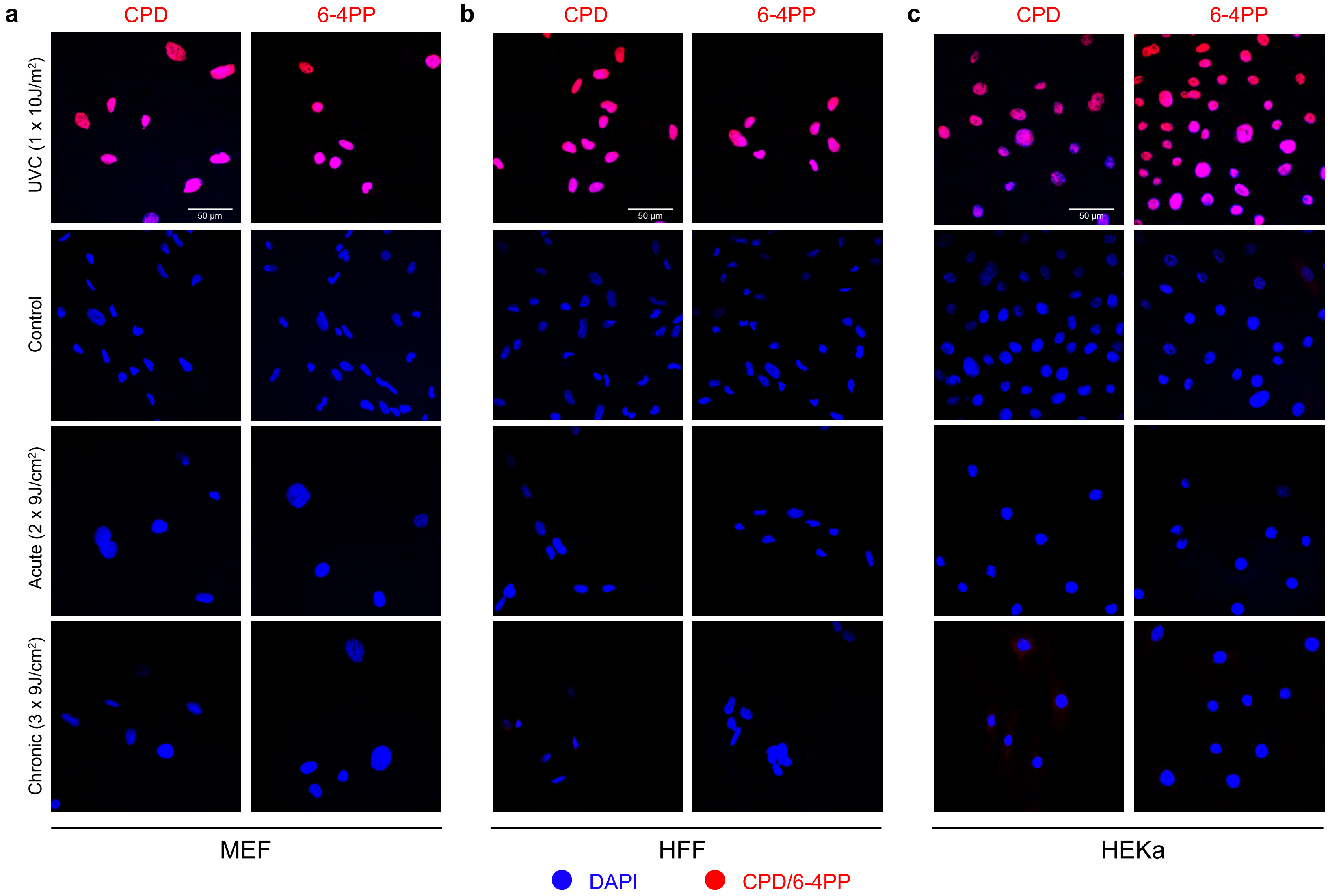

Supplementary Figure 2. Evaluation of cyclobutane pyrimidine dimers and 6-4 photoproducts irradiated by a UV-nail dryer. ***(a-c)*** Immunofluorescence of cyclobutane pyrimidine dimers (CPDs) and 6-4 photoproducts (6-4PPs) in MEF ***(a)***, HFF ***(b)***, and HEKa ***(c)*** cells after acute

or chronic irradiation with a UV-nail dryer. DAPI is shown in blue while CPDs and 6-4PPs are shown in red. A positive control, namely, cells irradiated with ultraviolet C (UVC) light (1x 10J/m^2), is also provided for each cell line. Images collected from four independent experiments for MEFs and HFFs and five independent experiments for HEKa cells.

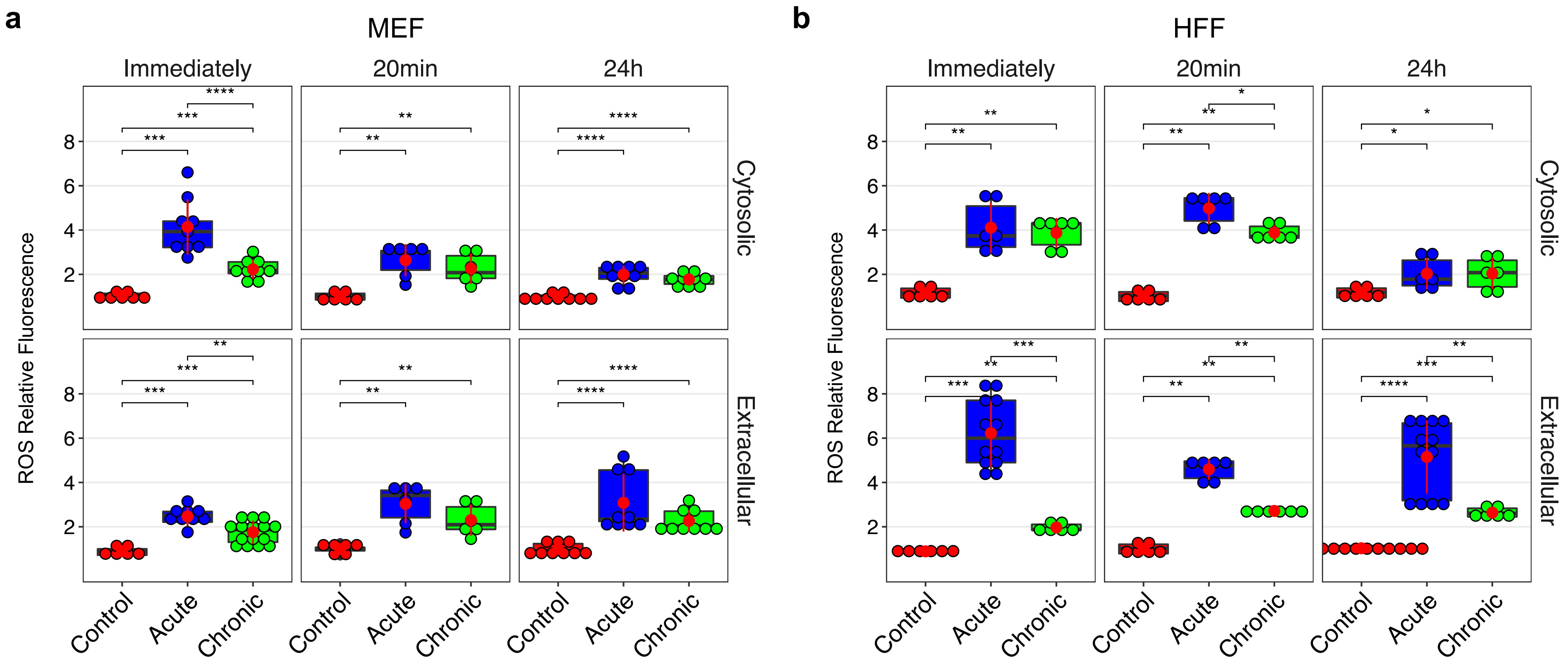

Supplementary Figure 3. Cytosolic and extra-cellular measurement of reactive oxygen species in MEFs and HFFs. Accumulation of reactive oxygen species (ROS) through time in primary MEFs ***(a)*** and HFFs ***(b)*** after acute or chronic irradiation with a UV-nail dryer. Each circle corresponds to a single experiment for a particular exposure. All y-axes reflect, either cytosolic or extracellular, relative ROS fluorescence by normalizing the fluorescence of a particular exposure to the average fluorescence of the appropriate controls. Data collected from *n=9* independent experiments for MEFs (exceptions apply for all ROS measurements 20 minutes after exposure where *n=6*, and for ROS measurement extracellularly immediately after chronic exposure in MEFs and in control samples where *n=15*) and *n=6* independent experiments for HFFs (exception for ROS measurement extracellularly immediately and 24h after acute irradiation in HFFs and in control samples at 24h where the used number of independent biological replicates is *n=12*). The bounds of the boxplots represent the interquartile range divided by the median, and Tukey-style whiskers extend to a maximum

of 1.5 × interquartile range beyond the box. Statistically significant results from FDR corrected Mann-Whitney U two-sided tests are denoted as: **q-value<0.01, ***q-value<0.001 and ****q-value<0.0001.

a

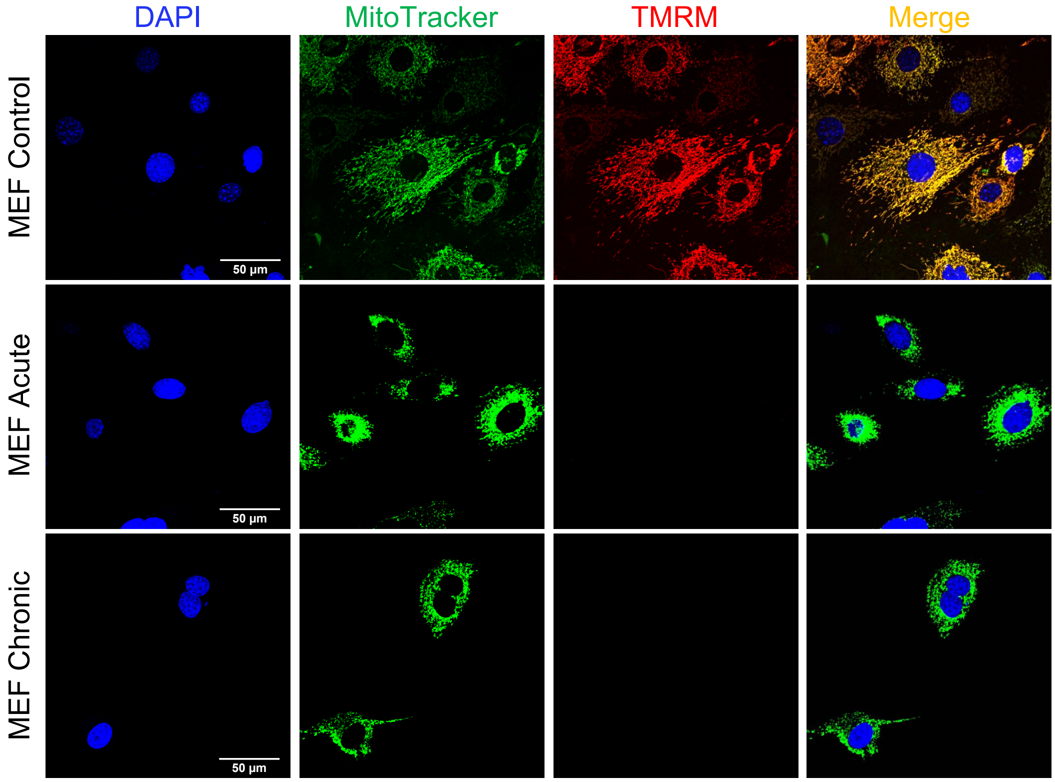

b

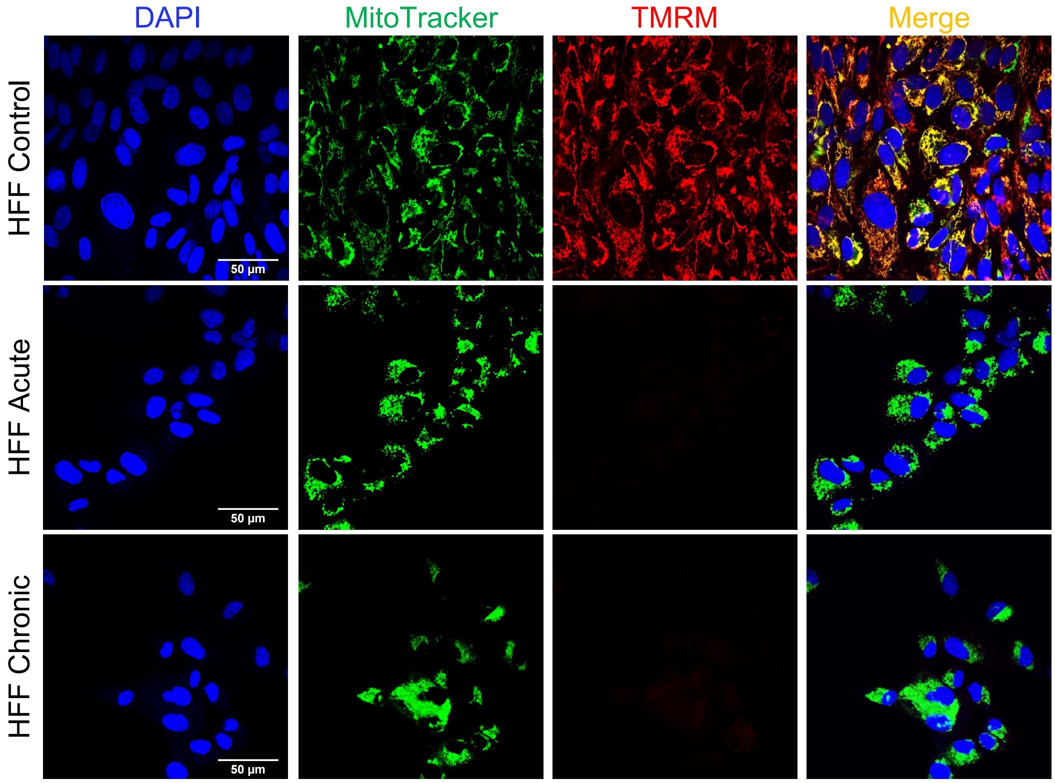

Supplementary Figure 4. Mitochondrial membrane potential after irradiation with a UV-nail dryer. Evaluating the mitochondrial membrane potential after irradiation of MEF cells ***(a)*** and HFF cells ***(b)*** with a UV-

nail dryer. DAPI is shown in blue, MitoTracker in green, and tetramethylrhodamine dye (TMRM) in red. Yellow corresponds to overlaps between MitoTracker and TMRM. Images collected from three independent experiments for MEFs and HFFs.

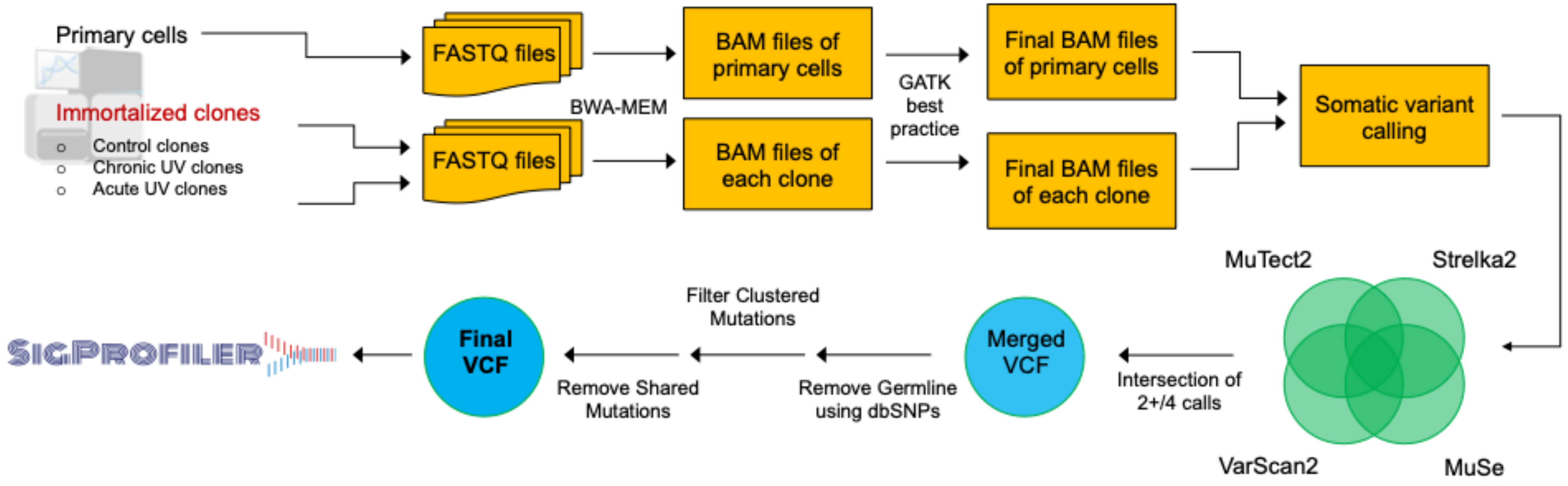

Supplementary Figure 5. Schematic of whole-genome data analysis. Primary cells were sequenced and used as a germline reference. We employed three variant callers (Mutect2, VarScan2, Strelka2, and MuSe) in matched tumor-normal mode, with the primary cells' BAM files as normal for each cell line. We selected variants called in at least 2 of the variant callers in order to increase variant calling quality. Germline single nucleotide polymorphisms (SNP) were removed using the dbSNP database. Any clustered mutation as well as any mutation observed in 2 or more samples were also removed. The final set of somatic mutations were analyzed by the SigProfiler suite of tools.

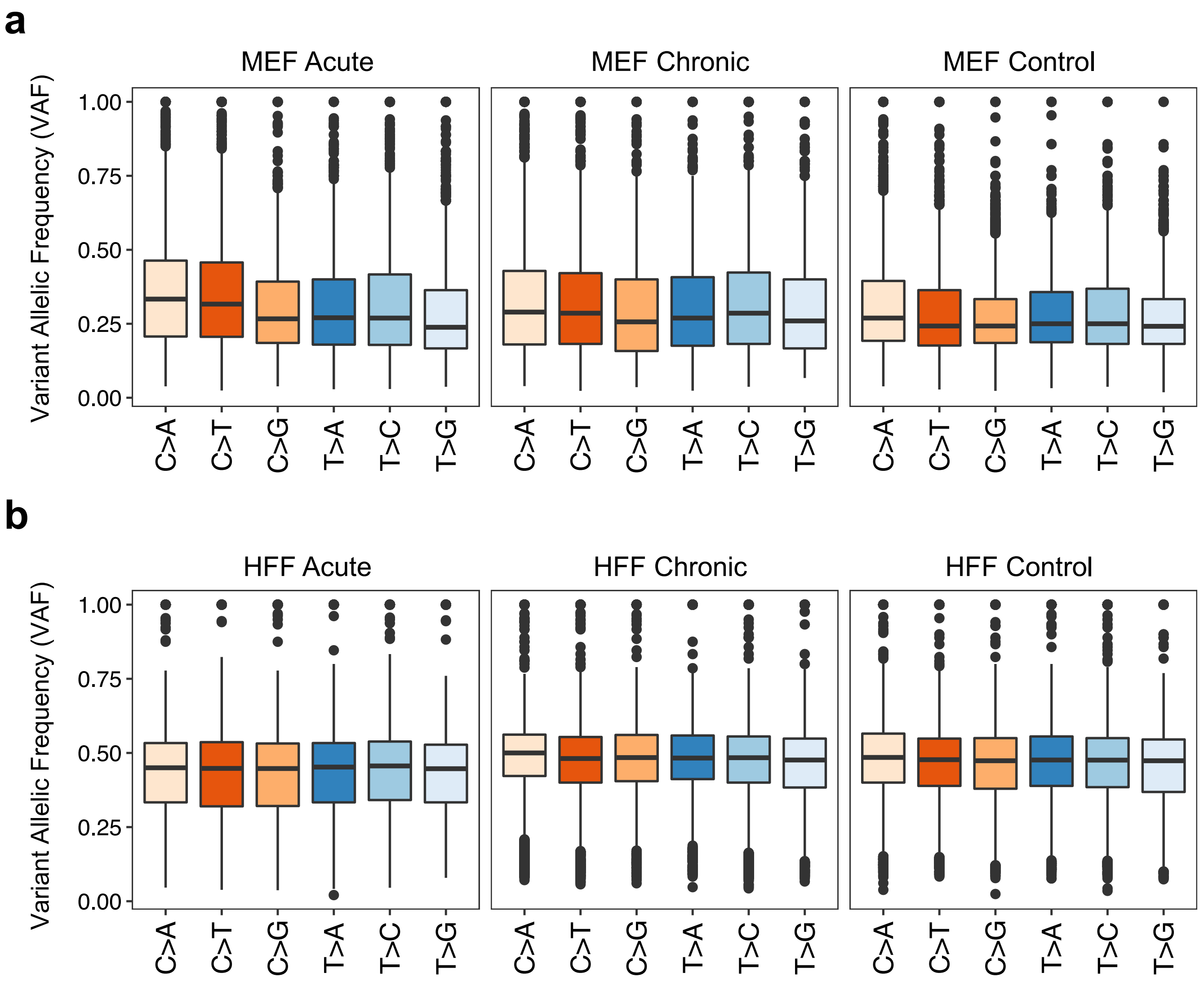

Supplementary Figure 6. Analysis of variant allelic frequency analysis. Variant allelic frequency of *bona fide* mutations in MEF clones ***(a)*** HFF clones ***(b)*** for each irradiation condition. Data collected from *n=5* independent biological replicates for each condition in MEFs and HFFs. The bounds of the boxplots represent the interquartile range divided by the median, and Tukey-style whiskers extend to a maximum of 1.5 × interquartile range beyond the box.

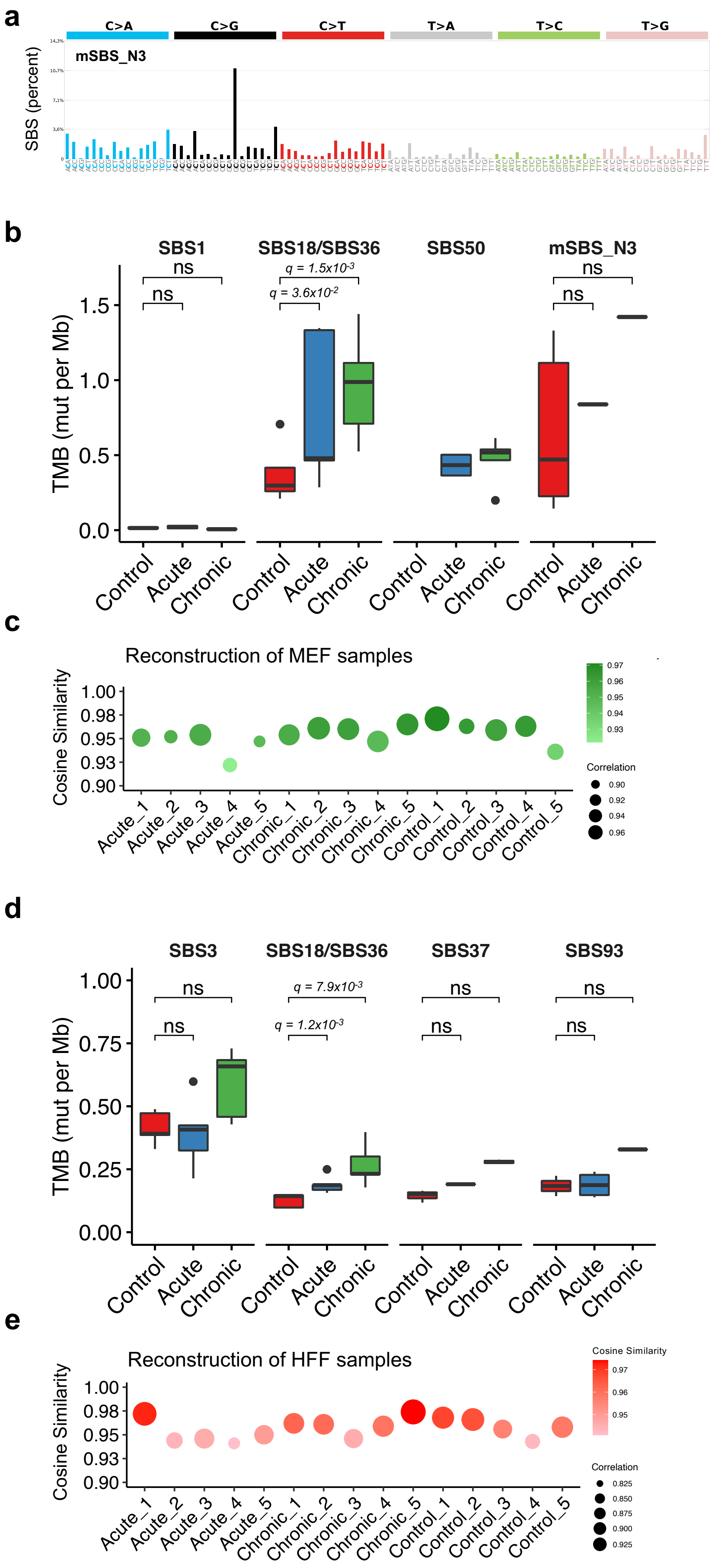

Supplementary Figure 7. Mutation analysis of UV-treated MEF and HFF clones and PCAWG samples. ***(a)*** Mutational signature mSBS_N3 identified *in vivo* in mouse models. ***(b)*** COSMIC signatures assigned to MEF clones per condition (control, acute and chronic). Data collected from *n=5* independent biological replicates for each condition. Statistically significant results from FDR corrected Mann-Whitney U two-sided tests are denoted as *q*-values. (***c***) Mutational signatures reconstruction of MEF mutational patterns. Accuracy is measured in cosine similarity between the original pattern of a sample and the pattern of a sample reconstructed using the assigned mutational signatures. (***d***) COSMIC signatures assigned to HFF clones per condition (control, acute and chronic). Data collected from *n=5* independent biological replicates for each condition. Statistically significant results from FDR corrected Mann-Whitney U two-sided tests are denoted as *q*-values. (***e***) Mutational signatures reconstruction of HFF mutational patterns. The bounds of the boxplots represent the interquartile range divided by the median, and Tukey-style whiskers extend to a maximum of 1.5 × interquartile range beyond the box.

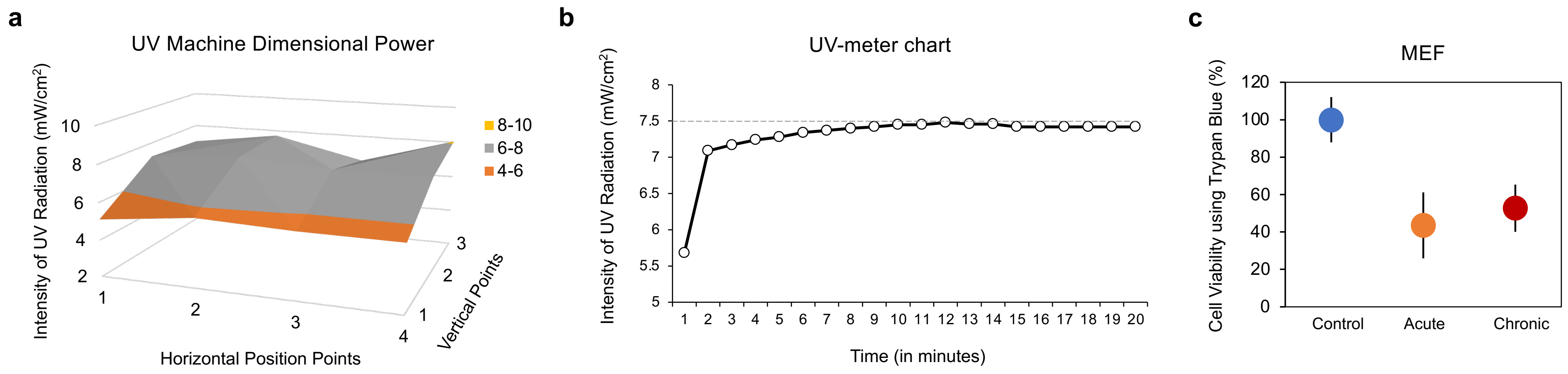

Supplementary Figure 8. Toxicity and characteristics of the UV-nail polish dryer machine. ***(a)*** Intensity of UV radiation (mW/cm^2) at different position points (vertical and horizontal) in the employed UV nail polish dryer. ***(b)*** Stability assessment of the UV intensity emitted from the UV-nail polish dryer over time. ***(c)*** Validation of the half maximal inhibitory concentration (IC50) used for the acute and chronic exposure protocols in MEFs. Data presented as mean viability percent ± standard deviation from *n=3* independent biological replicates.

SUPPLEMENTARY TABLES

Supplementary Table 1. Assays and timepoints for assessing DNA damage. The table denotes timepoints at which DNA damage and other experimental assessments were performed for each condition in primary cell models, including interrogation of cytotoxicity, genotoxicity, oxidative damage, and mitochondrial damage.

Assessment	Assays	Timepoints
Cytotoxicity	Trypan Blue	48 hours
Cytotoxicity	Cell Counting kit -8	49 hours
Genotoxicity	ɣH2Ax immunofluorescence	4 hours
Genotoxicity	CPD/6-4PP immunofluorescence	Immediately
Oxidative Damage	CellROX green reagent	Immediately
Oxidative Damage	OxiSelect In vitro ROS/RNS assay kit	Immediately
Oxidative Damage	OxiSelect In vitro ROS/RNS assay kit	20 minutes
Oxidative Damage	OxiSelect In vitro ROS/RNS assay kit	24 hours
Oxidative Damage	EpiQuik 8-OHdG DNA damage quantification kit	Immediately
Oxidative Damage	EpiQuik 8-OHdG DNA damage quantification kit	4 hours
Oxidative Damage	EpiQuik 8-OHdG DNA damage quantification kit	24 hours
Mitochondrial Damage	TMRM mitochondrial membrane potential indicator	24 hours
Mitochondrial Damage	MitoSOX mitochondrial superoxide indicator	25 hours
