# Supplementary material for: DNA damage and somatic mutations in mammalian cells after irradiation with a nail polish dryer
Source: Nat Commun. 2023 Jan 17;14:276. doi: 10.1038/s41467-023-35876-8 (PMC9845303; doi:10.1038/s41467-023-35876-8)
Supplement: Supplementary file 2 — Reporting summary [file 41467_2023_35876_MOESM2_ESM.pdf]

nature portfolio

Corresponding author(s): Ludmil B. Alexandrov

Last updated by author(s): Nov 16, 2022

Reporting Summary

Nature Portfolio wishes to improve the reproducibility of the work that we publish. This form provides structure for consistency and transparency in reporting. For further information on Nature Portfolio policies, see our Editorial Policies and the Editorial Policy Checklist.

Statistics

For all statistical analyses, confirm that the following items are present in the figure legend, table legend, main text, or Methods section.

n/a	Confirmed	
☐	☒	The exact sample size (*n*) for each experimental group/condition, given as a discrete number and unit of measurement
☐	☒	A statement on whether measurements were taken from distinct samples or whether the same sample was measured repeatedly
☐	☒	The statistical test(s) used AND whether they are one- or two-sided<br>*Only common tests should be described solely by name; describe more complex techniques in the Methods section.*
☐	☒	A description of all covariates tested
☐	☒	A description of any assumptions or corrections, such as tests of normality and adjustment for multiple comparisons
☐	☒	A full description of the statistical parameters including central tendency (e.g. means) or other basic estimates (e.g. regression coefficient) AND variation (e.g. standard deviation) or associated estimates of uncertainty (e.g. confidence intervals)
☐	☒	For null hypothesis testing, the test statistic (e.g. *F*, *t*, *r*) with confidence intervals, effect sizes, degrees of freedom and *P* value noted<br>*Give P values as exact values whenever suitable.*
☒	☐	For Bayesian analysis, information on the choice of priors and Markov chain Monte Carlo settings
☒	☐	For hierarchical and complex designs, identification of the appropriate level for tests and full reporting of outcomes
☐	☒	Estimates of effect sizes (e.g. Cohen's *d*, Pearson's *r*), indicating how they were calculated

Our web collection on statistics for biologists contains articles on many of the points above.

Software and code

Policy information about availability of computer code

Data collection	In addition to the generate genomics data, data were collected from Pancancer Analysis of Whole Genomes (PCAWG) of the International Cancer Genome Consortium (ICGC) based on their official data release.<br>Somatic mutations in whole-genome sequencing data were identified using our ensemble variant calling pipeline, which is freely available under the permissive 2-clause BSD license at: https://github.com/AlexandrovLab/EnsembleVariantCallingPipeline. All other computational tools utilized in this publication have been previously published and can be access through their respective publications:<br>BWA (v.0.7.17), GATK4 (v.4.1.4.1-0), MuSE (v.1.0), Strelka (v.2.9.10), VarScan (v2.4.3), VEP (Ensembl API 96).
Data analysis	R (v.3.6.0 to v.4.2.0), samtools (v.1.9), BWA (v.0.7.17), VEP (Ensembl API 96), IGV (v.2.6.2).<br><br>R packages: ggplot2 (v.3.3.6), easyGgplot2 (v.1.0.0.9000), ComplexHeatmap (v.2.12.0), circlize (v.0.4.15), ggpubr (v.0.4.0), corrr (v.0.4.3), lsa (v.0.73.3). In addition, RColorBrewer, brew, reshape2, dplyr, plyr, GenomicRanges, stringr, tidyr, tidyverse, hrbrthemes, data.table, readxl.<br><br>Fluorescence imaging: Fiji software (version 2.3.0), Nikon WorkStation 5317.

For manuscripts utilizing custom algorithms or software that are central to the research but not yet described in published literature, software must be made available to editors and reviewers. We strongly encourage code deposition in a community repository (e.g. GitHub). See the Nature Portfolio guidelines for submitting code & software for further information.

Data

Policy information about availability of data

All manuscripts must include a data availability statement. This statement should provide the following information, where applicable:

- Accession codes, unique identifiers, or web links for publicly available datasets
- A description of any restrictions on data availability
- For clinical datasets or third party data, please ensure that the statement adheres to our policy

All whole-genome and duplex sequencing data have been deposited to Sequence Read Archive (SRA) and can be downloaded using accession number: PRJNA667106. For mouse samples, we used the GRCm38 reference genome. For human samples, we used the GRCh38 reference genome. We employed the dbSNP142 for germline mutations detection in mouse samples, and dbSNP155 for human samples.

Human research participants

Policy information about studies involving human research participants and Sex and Gender in Research.

Reporting on sex and gender	N/A
Population characteristics	N/A
Recruitment	N/A
Ethics oversight	N/A

Note that full information on the approval of the study protocol must also be provided in the manuscript.

Field-specific reporting

Please select the one below that is the best fit for your research. If you are not sure, read the appropriate sections before making your selection.

☒ Life sciences ☐ Behavioural & social sciences ☐ Ecological, evolutionary & environmental sciences

For a reference copy of the document with all sections, see nature.com/documents/nr-reporting-summary-flat.pdf

Life sciences study design

All studies must disclose on these points even when the disclosure is negative.

Sample size	Every experiment was performed with at least three replicates with most experiments having five or more independent replicates. Three replicates were used for statistical comparisons using Student's t-tests. Sample size of five replicates was chosen to allow using the Mann-Whitney-U tests for most comparisons. The utilized sample sizes allowed detecting as low as 1.20-fold enrichments and depletions.
Data exclusions	No data were excluded from the analyses.
Replication	Every experiment was performed with at least three replicates. All replications were successful.
Randomization	Samples were allocated in groups based on irradiation parameters. Comparisons were performed between different types of irradiated samples and control samples.
Blinding	Blinding is not relevant for this study and, to the best of our knowledge, is never utilized when performing genotoxicity and mutagenicity testing.

Reporting for specific materials, systems and methods

We require information from authors about some types of materials, experimental systems and methods used in many studies. Here, indicate whether each material, system or method listed is relevant to your study. If you are not sure if a list item applies to your research, read the appropriate section before selecting a response.

Materials & experimental systems

n/a	Involved in the study
☐	☒ Antibodies
☐	☒ Eukaryotic cell lines
☒	☐ Palaeontology and archaeology
☒	☐ Animals and other organisms
☒	☐ Clinical data
☒	☐ Dual use research of concern

Methods

n/a	Involved in the study
☒	☐ ChIP-seq
☒	☐ Flow cytometry
☒	☐ MRI-based neuroimaging

Antibodies

Antibodies used

Primary antibodies used: Ser139-phosphorylated H2Ax (gH2Ax) (9718, Cell Signaling Technology), anti-cyclobutane pyrimidine dimers (CPDs) monoclonal antibodies (clone KTM53, Kamiya Biomedical) and anti-6-4 photoproducts monoclonal antibodies (Clone 64M-2, Cosmo Bio).

Secondary antibodies used: A fluorochrome-conjugated anti-rabbit secondary antibody (4412, Cell Signaling Technology), and a fluorochrome-conjugated anti-mouse Alexa Fluor 594 secondary antibody (8890S, Cell Signaling Technology).

Validation

Ser139-phosphorylated H2Ax (gH2Ax) (9718, Cell Signaling Technology) - According to manufacturer's protocol, antibody has been validated on human and mouse samples. Company website reports high specificity and rigorously validation in-house.

Anti-cyclobutane pyrimidine dimers (CPDs) monoclonal antibodies (clone KTM53, Kamiya Biomedical) - According to manufacturer's protocol, antibody has been validated on human and mouse samples. Company website reports several publications where the antibody was validated to react specifically with thymine dimers produced by UV irradiation in double- or single-stranded DNA, and does not react with (6-4) photo products.

Anti-6-4 photoproducts monoclonal antibodies (Clone 64M-2, Cosmo Bio) - According to manufacturer's protocol, antibody has been validated on human and mouse samples. Company website reports twelve publications where the antibody was validated to have high sensitivity detection of 6-4PPs in DNA purified from cultured cells.

Eukaryotic cell lines

Policy information about cell lines and Sex and Gender in Research

Cell line source(s)

Primary mouse embryonic fibroblasts (MEFs) from Lonza (M-FB-481); Primary human cells derived from human foreskin fibroblasts (HFFs) from Dr. John Murray (Indiana Universitt Bloo); Primary human keratinocytes, derived from normal adult human epidermal keratinocytes (HEKa) from ATCC (PCS-200-011; Lot number: 70033063).

Authentication

MEFs were authenticated by PCR of the short tandem repeats (STR) to confirm the correct species and that they are contamination-free. HFFs were authenticated by PCR of the short tandem repeats (STR) to confirm the correct species and that they are contamination-free. HFFs were authenticated by PCR of the short tandem repeats (STR) to confirm the correct species and that they are contamination-free.

Mycoplasma contamination

All cell cultures were bi-weekly tested for the absence of mycoplasma. Our cultures were negative.

Commonly misidentified lines
(See ICLAC register)

No commonly misidentified cell lines were used in this study.
